# Using probiotics to improve the utilization of chopped dried date palm leaves as a feed in diets of growing Farafra lambs

**DOI:** 10.3389/fvets.2022.1048409

**Published:** 2022-10-26

**Authors:** Hatem A. Hamdon, Ayman Y. Kassab, Einar Vargas-Bello-Pérez, Galal A. Abdel Hafez, Talaat A. Sayed, Mohsen M. Farghaly, Ahmed E. Kholif

**Affiliations:** ^1^Department of Animal Production, Faculty of Agriculture, New Valley University, Kharga, Egypt; ^2^Department of Animal Sciences, School of Agriculture, Policy and Development, University of Reading, Reading, United Kingdom; ^3^Department of Animal Production, Faculty of Agriculture, Assiut University, Assiut, Egypt; ^4^Department of Dairy Science, National Research Centre, Giza, Egypt

**Keywords:** blood metabolites, date palm leaves, digestibility, performance, wheat straw

## Abstract

The study determined the ability of three probiotics to improve the nutritional value of date palm leaves in diets of growing lambs. Twenty male Farafra lambs (26 ± 0.33 kg) were randomly allocated to one of four treatments (*n* = 6) and fed: a control or basal diet (C; 70% concentrate + 30% date palm leaves without additives) and supplemented with Bacillofort containing 2 × 10^11^ CFU of *Bacillus subtilis*/g (BAC treatment), Lacotpro containing 1 × 10^12^ CFU of *Lactobacillus acidophilus*/g (LAC treatment) or ZAD containing 6 × 10^8^ CFU of *R. albus*/g (ZAD treatment) at 4 g of all additives for 150 days. As a result of this study, LAC improved (*P* < 0.05) growth performance and feed efficiency compared to control. Additives increased (*P* = 0.001) concentrations of albumin, triiodothyronine, and thyroxine, hemoglobin concentration and red blood cells and decreased (*P* = 0.001) globulin and urea-N. Additives increased hot carcass (*P* = 0.040) while BAC increased *Longissimus dorsi*, meat and fat without affecting water holding capacity compared to other treatments. In the metabolism experiment, BAC increased the digestibility of crude protein, while BAC and ZAD increased the digestibility of dry matter, organic matter, and neutral detergent fiber. Additives did not affect nitrogen (N) intake and urinary N; however, decreased fecal N and increased N balance compared to the control. BAC and ZAD increased ruminal volatile fatty acids concentration compared to the control. Based on our results, Lacotpro could be used to improve growth performance and feed efficiency, while Bacillofort could be used to improve meat quality of in lambs.

## Introduction

Semi-arid and arid areas have difficult conditions including low rainfall, resulting in low livestock production with low quality forages (1) making trees and shrubs as sources of small ruminants feed (2). Moreover, in many countries, including Egypt, there is a shortage of feed availability, making the use of unusual feeds and agricultural byproducts as critical options for animal feed (3). The main challenge of using such resources is their low nutritive value (4). Thus, enhancing the nutritive value of fibrous feed is essential (5).

Date palm (Phoenix dactilifera) is an important crop in Egypt, with roughly 650,000 tons of dry matter (DM) annually (6). Approximately 20 kg DM are produced from each palm tree, without a significant utilization. The major challenges of feeding date palm byproducts (i.e., leaves) are the high levels of neutral detergent fiber (NDF; 58%), low levels of crude protein (CP; 5%) and low digestibility (< 50%) compared to other forage feeds (7, 8). Recently, Kholif et al. (7) evaluated the nutritive value of date palm leaves and reported its potential as a sustainable feed for ruminant. With lactating Farafra animals, Kholif et al. (1, 5) observed improved feed utilization and lactation performance when fed ewes on a diet based on date palm leaves and treated with organic acids, fibrolytic enzymes and multi-species probiotics. Feeding some microbes proved to be a beneficial strategy to improve utilization of low-quality feeds and animal performance (1).

In growing animal diets, probiotic additives may offer alternatives to ionophores (9). Probiotics alter rumen microflora populations and fermentation end-products (10–12). Reducing animal health problems is associated with improvements in immunological responses which reduces the need for the use of antimicrobials, and in turn boost growth performance and productive efficiency (11–13). The main problem with probiotic administration in animal diets is the high variability between experiments and, its efficacy depends on the specific product and concentration (14, 15). In many experiments, probiotics improved nutrient absorption and reduced pathogens counts (16), as well as enhanced immune system and competed with invading microbes without leaving any residual toxic effects in an eco-friendly manner (17). Sallam et al. (18) reported that feeding microbial feed additives to growing Barki lambs fed diets that included peanut hay, resulted in improved fiber digestibility, N intake and N retention and enhanced final body weight gain without affecting feed efficiency. Moreover, Hamdon et al. (2) fed Farafra lambs on date palm-based diet supplemented with Bacillus subtilis and Phanerochaete chrysosporium and observed improved growth performance and feed utilization.

However, many experiments evaluated the inclusion of probiotics in diets of animals, the present study evaluated three local developed products with an ingredient (i.e., date palm leaves) that has gained increasing interests in recent years. Therefore, we hypothesized that feeding bacterial feed additives would affect ruminal environment and improve feed digestion resulting in improved performance of growing Farafra lambs. Accordingly, the present study aimed to evaluate the effects of three bacterium containing products in the diet of Farafra lambs fed a control diet based on palm leaf hay. Two experiments were performed: (i) to determine the effects of diets on feed intake, performance efficiency, blood metabolites and carcass characteristics of the growing Farafra lambs, and (ii) to determine the effects of diets on nutrient digestibility of adult Farafra rams.

## Materials and methods

This study was performed at the Department of Animal Production, Faculty of Agriculture, New Valley University, New Valley, Egypt (25°26′N and 30°32′E). The area has a desert climate with rare rains (0.18 rainy days, 0.05% of the time), 0.08 millimeters of precipitation and high temperature (23°C during January and 41°C during June to August).

### Microbial products

Three probiotics were used in this study: (i) Bacillofort (Bactizad, Al Sharqia, Egypt) is a microbial feed additive that contains 2 × 10^11^ CFU of *B. subtilis*, 100 g mannan oligosaccharides, 150 g of β-glucan and corn starch as a carrier. (ii) Lacotpro (Bactizad, Al Sharqia, Egypt) is a microbial feed additive that contains 1 × 10^12^ CFU of *Lactobacillus acidophilus*, 100 g mannan oligosaccharides, 150 g β-glucan and corn starch as a carrier. (iii) ZAD (Bactizad, Al Sharqia, Egypt) is microbial feed additive that contains 6 × 10^8^ CFU of *Ruminococcus albus*, 100 g mannan oligosaccharides, 150 g of β-glucan and corn starch as a carrier.

### Experiment 1 (growth performance)

Twenty Farafra lambs with an average body weight of 26 ± 0.33 kg and 5.5 months of age were randomly assigned to four experimental groups (5 lambs per treatment) and fed according to their average initial weights. The experimental period lasted 150 d, consisting of a 15-d adaptation period followed by a 150-d sampling period. Lambs were offered a basal diet based on concentrate and ground wheat straw at 70:30 without additives (control treatment) or supplemented with Bacillofort (BAC treatment), Lacotpro (LAC treatment) or ZAD (ZAD treatment) at 4 g/lamb daily. The concentrate feed mixture contained 500 g corn, 320 g wheat bran, 150 g soybean meal, 20 g limestone, 5 g sodium chloride, and 5 g minerals and vitamins mixture per kg DM. Diets were prepared to meet the nutrient needs of lambs according to the NRC (19) combined with a 10% margin to ensure collection of orts. Ingredients were mixed and the whole concentrate was analyzed (Table 1). Lamb's needs were corrected once every 2 weeks based on changes in body weight. Diets were offered to each lamb individually at 08:00 and 16:00 h in two equal portions. Diets were prepared, mixed 1 day before feeding. Daily allocations of probiotics for each lamb were manually provided individually in 100 g of concentrate before morning feeding at 08:00 h to assure intake. Samples of feed ingredients were obtained daily, composited weekly, and dried at 60 °C in a forced-air oven for 48 h.

**Table 1:** Chemical composition (g/kg DM) of date palm leaves, the concentrate feed mixture and basal diet used in experiment 1 and 2.

**Items**	**Diet^a^**	**CFM^b^**	**Date palm leave**
Dry matter	938	656	940
Organic matter	911	653	861
Crud protein	156	143	52.0
Ether extract	58.4	49.4	30.1
Nonstructural carbohydrates	306	260	154
Neutral detergent fiber	388	201	624
Acid detergent fiber	334	184	500
Hemicellulose	53.9	16.6	125
Cellulose	218	104	381
Fe (mg/kg)	Not determined	Not determined	151
Zn (mg/kg)	Not determined	Not determined	139
Se (mg/kg)	Not determined	Not determined	3.93
Cu (mg/kg)	Not determined	Not determined	30.2

^a^Control diet contained 700 g of concentrate feed mixture and 300 g of date palm leaves.

^b^The concentrate feed mixture (CFM) consisted of (per kg DM): 500 g corn, 320 g wheat bran, 150 g soybean meal, 20 g limestone, 5 g sodium chloride, and 5 g minerals and vitamins mixture (containing per kg: 141 g Ca, 87 g P, 45 g Mg, 14 g S, 120 g Na, 6 g K, 944 mg Fe, 1,613 mg Zn, 484 mg Cu, 1,748 mg Mn, 58 mg I, 51 mg Co, 13 mg Se, 248,000 IU vitamin A, 74,000 IU vitamin D3, 1,656 IU vitamin E).

During sampling, feed intake was recorded as the difference between feed offered and refusals from the previous day. Lambs were biweekly weighed, and feed efficiency was calculated as g of feed per g of weight gain.

As previously reported by Kholif et al. (20), blood samples (about 10 mL) were collected from all the lambs from the jugular vein on days 30, 60, 90, 120 and 150 after the morning feeding at 10:00 h. The last sample was obtained at the time of slaughter at 150 d. Blood samples were immediately centrifuged at 3,000 × g for 15 min, and serum samples were stored at −20 °C until analysis. Stored serum samples were analyzed for total protein, albumin, glucose, urea-N, creatinine, total cholesterol, alanine aminotransferase (ALT), aspartate aminotransferase (AST) cholesterol, triglyceride, triiodothyronine (T3), thyroxine (T4) and globulin as reported previously (2).

As previously reported by Rivero et al. (21), the hematological parameters were assessed by using automatic, fully Digital Hematology Analyzer (Shenzhen Mind ray Auto Hematology Analyzer (Model Bc-3200, Shenzhen Mind ray Biomedical Electronics Co. Hamburg 20,537, Germany). These parameters included a total count of white blood cells (WBC), the total count of red blood cells (RBC), hemoglobin, hematocrit (HCT), mean corpuscular hemoglobin (MCH), mean corpuscular volume (MCV), and mean corpuscular hemoglobin concentration (MCHC).

At the end of the experimental period, three random animals from each group were slaughtered. Fasting body weight was recorded and immediately after slaughter, the hot carcass weight was recorded, and the weights of the head, pelt, liver, lungs, heart, spleen, kidneys, kidney fat, omental fat, testes, trachea, and heart were recorded. Full methodological details for charctass traits have been reported in a companion paper (2).

Water holding capacity (WHC) was measured by weighing about 0.3 g (W1) of meat in a filter paper (Whatman No.1) and subjected to pressure of 1,000 gm for 10 min, then it was weighed again (W2). WHC was estimated as a percentage according to the following formula: WHC % = [W2/W1] × 100. WHC was expressed as the percentage of loss related to the initial weight (22).

### Experiment 2 (digestibility experiment)

As in Experiment 1, 12 adult rams weighing 50 ± 0.25 kg were randomly allocated into four treatment groups to determine the nutrient digestibility using the fecal bag technique. Animals received the same diets as in Experiment 1. Rams were housed individually in metabolic cages (70 cm width × 120 cm high × 150 cm length). The experiment lasted 22 d, consisting of a 15-d adaptation period followed by 7 d of sample collection. Feeds and orts also were dried before grinding through a 1-ml screen for chemical analysis. Feces were collected daily, and 10% of the fecal matter (by weight) was dried at 70 °C for 24 h, combined and ground through a 1-mm mill screen for subsequent chemical analysis. Apparent digestion coefficients of the nutrients were calculated by expressing the difference between the nutrient content in both consumed feed and feces as a percentage of intakes.

During the collection period, urine was also collected in large polyethylene bags containing 20 ml of 10% H_2_SO_4_ to prevent losses of ammonia N. Daily urine sample of 10% was collected for each ram and stored in a glass bottle in a refrigerator until it was used for nitrogen (N) determination to calculate the N balance.

Ruminal contents (about 150 mL) were monthly sampled at 3 h after the morning feeding to determine the pH and fermentation end-products. After initial discarding of 50 ml ruminal fluid to prevent contamination with saliva, 100 ml ruminal fluid were collected by utilizing a stomach tube, and samples removed from each lamb were strained through 4 layers of cheesecloth. Ruminal fluid pH was determined using a pH meter (Beckman, model 45, USA).

A subsample of 5 mL ruminal fluid was preserved with 5 mL of 0.2 M HCl for ammonia-N analysis (23), and 0.8 mL of ruminal fluid was mixed with 0.2 mL of a solution containing 250 g of metaphosphoric acid/L for total volatile fatty acids (VFA) analysis. Samples were preserved at −20°C until analyses (24).

Samples of fermented fluid (4 mL) were individually combined with 4 mL of methyl green-formalin-saline solution and stored in a refrigerator at 4°C until analysis of bacterial and protozoal count following methods from Dehority (25). Total bacteria concentration was determined using a Petroff-Hausser counting chamber (Hausser Scientific^®^, 3900, Horsham, PA) and a phase contrast microscope at a magnification of 100 ×.

The nutritive value of diets as total digestible nutrients (TDN), digestible energy (DE), metabolizable energy (ME) were calculated according to NRC (26) equations, while the unité fourragère du lait (UFL; net energy requirements for lactation equivalent to 1 kg of standard air-dry barley) was used according to INRA (27) equation.

Feed, orts, and fecal samples were ground through a 1-mm screen using a Wiley mill and analyzed for DM (ID 930·15), ash (ID 942·05), N (ID 954·01), and ether extract (EE) (ID 920·39), according to the official methods of the AOAC (23). NDF levels were determined by the procedure of Van Soest et al. (28) with sodium sulphite. Acid detergent fiber (ADF) concentrations (method ID 973·18) were analyzed as described by the AOAC (23). Non-structural carbohydrate [NSC = 1,000 – (NDF + CP + EE + ash)] and organic matter (OM = 1,000 – ash) concentrations were determined.

### Statistical analysis

Data for total gain, carcass characteristics and composition, and nutrient digestibility were analyzed using the PROC GLM procedure of SAS, online version (SAS Inst. Inc. Cary, NC, USA) for a complete randomized design with individual animal as the experimental unit. The model included the effect of treatment. Measurements (body weight changes by difference, feed intake and conversion, and blood measurements) recorded daily or weekly were analyzed as repeated measures. When the treatment *F*-test was significant at *P* < 0.05, means were compared by applying the probability of difference option of the least squares means statement. The treatment × week interaction was non-significant (i.e., *P* > 0.05) for most of the measurements; thus, only the main effects of treatments were reported.

## Results

### Feed intake, growth performance and feed efficiency

Probiotics did not impact feed intake (Table 2). Initial weight did not vary between treatments. However, LAC improved final body weight (*P* = 0.120), total weight gain (*P* = 0.042) and daily gain (*P* = 0.033), and feed efficiency (*P* = 0.011) while ZAD decreased these parameters compared to the control.

**Table 2:** Feed intake, growth performance, and feed efficiency of growing Farafra lambs fed diets containing date palm leaves treated with different probiotics.

	**Diet^1^**				**SEM**	***P* value**
	**Control**	**BAC**	**LAC**	**ZAD**		
Intake (g/d)	1,577	1,623	1,614	1,617	12.5	0.120
Initial weight (kg)	26.0	26.0	26.0	26.3	2.42	0.991
Final weight (kg)	53.2^b^	51.5^b^	57.5^a^	49.3^c^	2.06	0.011
Total weight gain (kg)	27.2^b^	25.5^c^	31.5^a^	23.0^c^	1.92	0.042
Daily gain (g/d)	181.1^b^	170.3^c^	2109.8^a^	1543.7^c^	4.01	0.033
Feed efficiency (g feed/g gain)	8.71^c^	9.53^b^	7.69^d^	10.52^a^	0.613	0.011

Means in the same row with different superscripts differ at P < 0.05. P-value is the observed significance level of the F-test for diet; SEM, standard error of the mean.

^1^Diet: Control diet contained 700 g of concentrate feed mixture and 300 g of date palm leaves without additives or supplemented with Bacillofort probiotic (BAC treatment), Lacotpro probiotic (LAC treatment) or ZAD probiotic (ZAD treatment).

### Blood measurements

Compared to control, additives did not impact blood glucose, AST, ALT, and triglyceride; however, additives increased (*P* = 0.001) concentrations of albumin, T_3_ and T_4_ and decreased (*P* = 0.001) globulin and urea-N (Table 3). BAC and ZAD increased concentrations of blood total proteins (*P* = 0.001) and decreased blood creatinine (*P* = 0.024). BAC and ZAD lowered (*P* = 0.031) cholesterol compared to control.

**Table 3:** Blood metabolites (g/dL) and hematological parameters of growing Farafra lambs fed diets containing date palm leaves treated with different probiotics.

	**Diet^1^**				**SEM**	***P* value**
	**Control**	**BAC**	**LAC**	**ZAD**		
Total protein	6.08^b^	6.53^a^	6.34^b^	6.49^a^	0.041	0.001
Albumin	2.74^b^	3.43^a^	3.33^a^	3.33^a^	0.244	0.001
Globulin	3.34^a^	3.10^b^	3.01^b^	3.17^b^	0.020	0.001
Glucose (mg/dL)	75.1	76.7	76.6	78.3	1.70	0.538
AST (units/L)	60.5	61.2	60.6	60.8	2.10	0.621
ALT (units/L)	19.3	20.0	19.4	19.9	1.09	0.821
Urea-N (mg/dL)	31.0^a^	27.8^b^	28.8^b^	28.3^b^	0.51	0.001
Creatinine (mg/dL)	1.33^a^	1.05^b^	1.34^a^	1.04^b^	0.154	0.024
Cholesterol (mg/dL)	1001^c^	110^b^	100^c^	118^a^	1.4	0.031
Triglyceride (mg/dL)	62.2	59.3	60.9	60.4	1.86	0.677
T_3_ (ng/mL)	1.41^b^	2.65^a^	2.44^a^	2.38^a^	0.114	0.001
T_4_ (μg/mL)	5.56^c^	7.61^a^	6.72^b^	7.36^a^	0.123	0.001
Hemoglobin	9.36^b^	10.52^a^	10.24^a^	10.33^a^	0.101	0.001
Hematocrit (%)	35.2	36.7	36.1	36.4	1.11	0.111
RBC (10^6^/μL)	9.64^b^	10.58^a^	10.41^a^	10.58^a^	0.095	0.001
WBC (10^3^/μL)	8.41	8.58	8.79	8.76	0.312	0.821
MCV (fl)	35.9	37.3	36.9	37.2	0.14	0.753
MCH (pg)	10.2^b^	11.1^a^	10.9^a^	11.0^a^	0.07	0.001
MCHC (g/dL)	31.1	32.1	32.0	32.1	0.88	0.193

ALT, Alanine aminotransferase; AST, Aspartate transaminase; MCH, Mean corpuscular hemoglobin; MCHC, Mean corpuscular hemoglobin concentration; MCV, Mean corpuscular volume; RBC, Red blood cells; T3, Triiodothyronine; T4, Thyroxine; WBC, White blood cells.

Means in the same row with different superscripts differ at P < 0.05. P-value is the observed significance level of the F-test for diet; SEM, standard error of the mean.

^1^Diet: Control diet contained 700 g of concentrate feed mixture and 300 g of date palm leaves without additives or supplemented with Bacillofort (BAC treatment), Lacotpro (LAC treatment) or ZAD (ZAD treatment) probiotics.

Additives did not alter blood HCT, WBC, MCV or MCHC (Table 3). Raised (*P* = 0.001) blood hemoglobin and RBC were observed with the additive treatments.

### Carcass characteristics and meat composition

Increased fasting weight (*P* = 0.011) and legs weight (*P* = 0.022), and lowered brisket (*P* = 0.01) and flank weights (*P* = 0.016) were observed with BAC and ZAD; however, all additives increased hot carcass (*P* = 0.040), shoulder weight (*P* = 0.021), loin weight (*P* = 0.017) and rack weight (*P* = 0.021) compared to the control (Table 4). The heaviest *Longissimus dorsi* muscle was observed with BAC.

**Table 4:** Carcass traits and edible and non-edible parts of growing Farafra lambs fed diets containing date palm leaves treated with different probiotics.

	**Diet^1^**				**SEM**	***P* value**
	**Control**	**BAC**	**LAC**	**ZAD**		
Carcass traits (kg)						
Fasting weight	49.3^c^	57.5^a^	51.5^c^	53.2^b^	0.22	0.011
Slaughter weight	47.3^c^	55.5^a^	49.5^bc^	51.2^b^	0.93	0.010
Hot carcass	24.5^c^	29.1^a^	26.6^b^	28.2^a^	0.84	0.040
Dressing percentage (%)	49.7^b^	49.2^b^	56.5^a^	50.0^b^	1.75	0.031
Shoulder weight	4.92^c^	5.99^a^	5.53^b^	5.89^a^	0.133	0.021
Legs weight	6.83^b^	7.89^a^	7.22^ab^	7.79^a^	0.282	0.022
Loin weight	2.26^c^	2.86^a^	2.59^b^	2.71^b^	0.153	0.017
Neck weight	2.06	2.21	2.08	2.19	0.141	0.890
Rack weight	6.24^c^	7.32^a^	6.90^b^	7.14^a^	0.283	0.021
Brisket weight	1.09^c^	1.35^a^	1.09^c^	1.27^b^	0.120	0.010
Flank weight	1.09^c^	1.50^a^	1.16^c^	1.26^b^	0.062	0.016
*Longissimus dorsi* muscle	613^b^	745^a^	623^b^	637^b^	12.0	0.001
Edible and non-edible parts (kg)						
Tail	3.05^c^	4.34^a^	3.32^b^	3.37^b^	0.42	0.010
Pelt	5.41^b^	6.34^a^	6.17^a^	6.22^a^	0.74	0.004
Gastrointestin-Full	8.06^c^	9.48^a^	8.45^c^	8.74^b^	1.27	0.001
Empty gastrointestinal tract	2.36^c^	3.36^a^	2.45^c^	2.85^b^	0.11	0.001
Head	3.54^c^	4.29^a^	3.83^b^	4.19^a^	0.25	0.010
Feet	1.16^c^	1.39^a^	1.26^b^	1.33^a^	0.06	0.037
Kidney fat (g)	188^c^	312^a^	228^b^	245^a^	5.2	0.036
Omental fat (g)	418^c^	528^a^	488^b^	523^a^	6.2	0.030
Liver (g)	690^b^	817^a^	775^a^	802^a^	7.8	0.039
Kidney (g)	107^c^	137^a^	120^b^	123^b^	4.4	0.024
Testes (g)	527^b^	567^a^	542^b^	447^c^	4.3	0.019
Spleen (g)	105^b^	135^a^	127^a^	127^a^	2.3	0.012
Heart (g)	277	282	272	278	8.3	0.980
Lungs and trachea (g)	562^c^	657^a^	603^b^	618^a^	8.0	0.003

Means in the same row with different superscripts differ at P < 0.05. P-value is the observed significance level of the F-test for diet; SEM, standard error of the mean.

^1^Diet: Control diet contained 700 g of concentrate feed mixture and 300 g of date palm leaves without additives or supplemented with Bacillofort (BAC treatment), Lacotpro (LAC treatment) or ZAD (ZAD treatment) probiotics.

Additives increased the weights of tail (*P* = 0.010), pelt (*P* = 0.004), head (*P* = 0.01), feet (*P* = 0.037), kidney fat (*P* = 0.036), omental fat (*P* = 0.03), liver (*P* = 0.039), kidney (*P* = 0.024), spleen (*P* = 0.012), lungs and trachea (*P* = 0.003) compared to the control (Table 4). BAC increased the weight of testes compared to other treatments.

BAC increased the weights of *Longissimus dorsi* muscle (*P* = 0.022), meat (*P* = 0.003) and fat (*P* = 0.039) without affecting bone weight or WHC (Table 5). LAC and ZAD decreased the proportion of crude fat (*P* = 0.02) without influencing crude protein or ash.

**Table 5:** Gross and chemical compositions, and physicochemical properties of carcass of growing Farafra lambs fed diets containing date palm leaves treated with different probiotics.

	**Diet^1^**				**SEM**	***P* value**
	**Control**	**BAC**	**LAC**	**ZAD**		
Gross composition (g)
Bone	1,412	137	147	148	7.3	0.940
Meat	263^b^	312^a^	273^b^	295^a^	2.5	0.003
Fat	208^b^	297^a^	203^b^	193^b^	4.4	0.039
Chemical composition of *Longissimus dorsi* muscle (%, DM basis)
Crude protein	668	666	681	669	20.5	0.960
Crude fat	237^a^	241^a^	208^b^	218^b^	5.9	0.020
Ash	28.3	27.3	27.7	28.0	0.3	0.930
Physicochemical properties
Water holding capacity	20.4	25.5	21.6	22.0	4.12	0.105

Means in the same row with different superscripts differ at P < 0.05. P-value is the observed significance level of the F-test for diet; SEM, standard error of the mean.

^1^Diet: Control diet contained 700 g of concentrate feed mixture and 300 g of date palm leaves without additives or supplemented with Bacillofort (BAC treatment), Lacotpro (LAC treatment) or ZAD (ZAD treatment) probiotics.

### Nutrient digestibility and nitrogen balance

However, BAC increased the digestibility of CP, while BAC and ZAD increased the digestibility of DM, OM and NDF (Table 6). All additives increased the digestibility of NSC; however, LAC did not change digestibility of DM, CP, or NDF compared to the control. Both of BAC and ZAD increased the digestible CP, TDN, DE, ME, NEL and UFL compared to the control treatment.

**Table 6:** Feed intake, nutrient digestibility, diet nutritive values and nitrogen (N) balance of adult Farafra rams fed with diets containing date palm leaves treated with different probiotics.

	**Diet^1^**	**SEM**	***P* value**
	**Control**	**BAC**	**LAC**	**ZAD**		
Intake (g/d)	1622	1614	1618	1622	18.2	0.888
Nutrient digestibility (g digested/kg ingested)
Dry matter	547^c^	590^a^	557^c^	577^b^	10.7	0.021
Organic matter	601^c^	650^a^	639^ab^	642^a^	1.4	0.003
Crude protein	611^b^	650^a^	632^b^	639^b^	2.5	0.011
Ether extract	858	894	864	886	3.2	0.748
Nonstructural carbohydrates	679^b^	742^a^	741^a^	737^a^	3.6	0.012
Neutral detergent fiber	516^b^	589^a^	524^b^	571^a^	5.7	0.032
Nutritive values						
Digestible crude protein	97.1^c^	103.2^a^	100.3^c^	101.4^b^	0.2	0.011
TDN (g/kg DM)_2_	604^c^	653^a^	622^c^	647^b^	4.2	0.013
DE (Mcal/kg DM)^2^	2.66^c^	2.88^a^	2.74^c^	2.85^b^	0.031	0.010
ME (Mcal/kg DM)^2^	2.69^c^	2.91^a^	2.77^c^	2.88^b^	0.020	0.011
UFL (Mcal/kg DM)^3^	2.39^c^	2.60^a^	2.47^c^	2.58^b^	0.024	0.013
Nitrogen (N) utilization (g/day)						
Total N intake	41.2	41	41.1	41.2	1.49	0.914
Fecal N	16.3^a^	14.4^b^	14.8^b^	14.9^b^	0.44	0.041
Urinary N					0.93	0.555
N balance	18.2^b^	19.9^a^	19.6^a^	19.8^a^	0.49	0.031

Means in the same row with different superscripts differ at P < 0.05. P-value is the observed significance level of the F-test for diet; SEM, standard error of the mean.

^1^Diet: Control diet contained 700 g of concentrate feed mixture and 300 g of date palm leaves without additives or supplemented with Bacillofort (BAC treatment), Lacotpro (LAC treatment) or ZAD (ZAD treatment) probiotics.

^2^TDN = total digestible nutrients, DE = Digestible energy, ME = Metabolizable energy. All have been calculated according to NRC (26) equation.

^3^UFL = unité fourragère du lait (net energy requirements for lactation equivalent of 1 kg of standard air-dry barley) calculated according to INRA (27) equation.

Compared to control, additives did not influence N intake or urinary N; however, decreased fecal N and increased N balance (Table 6).

### Ruminal fermentation

Additives did not change ruminal pH or ammonia-N (Table 7). Both of BAC and ZAD increased ruminal VFA compared to the control.

**Table 7:** Ruminal fermentation of adult Farafra rams fed with diets containing date palm leaves treated with different probiotics.

	**Diet^1^**				**SEM**	***P* value**
	**Control**	**BAC**	**LAC**	**ZAD**		
pH	6.84	6.91	7.04	6.84	0.588	0.623
Volatile fatty acids (mmol/L)	60.5^c^	74.4^a^	62.8^c^	66.5^b^	1.40	0.021
Ammonia-N (mg/dL)	29.1	28.2	28.8	28.5	2.10	0.442
Protozoa count (× 10^5^ cell/mL)	4.60	4.78	4.22	4.39	0.514	0.313

Means in the same row with different superscripts differ at P < 0.05. P-value is the observed significance level of the F-test for diet; SEM, standard error of the mean.

^1^Diet: Control diet contained 700 g of concentrate feed mixture and 300 g of date palm leaves without additives or supplemented with Bacillofort (BAC treatment), Lacotpro (LAC treatment) or ZAD (ZAD treatment) probiotics.

## Discussion

The inclusion of probiotics in animal diets has been evaluated in many experiments. The novelty in the present study is the evaluation of three local developed probiotics added to diets containing date palm leaves that recently gained increasing interests in the sustainable sheep nutrition under desert and tropical conditions. More experiments are required to explore the effects of these probiotics in animal diets to explore their modes of action in the gastrointestinal tract of ruminants.

### Feed intake, growth performance and feed efficiency

Microbial feed additives did not affect feed intake and that somehow explains that there was unchanged feed palatability. In a previous experiment under the same environmental conditions, Hamdon et al. (2) observed that feeding *B. subtilis* and *P. chrysosporium* to growing Farafra lambs had weak effects on feed intake. However, others (18) observed increased feed intake when lambs were fed diets based on peanut hay supplemented with probiotics. Animal used, growth stage, environmental condition, dose, strains, duration and frequency or compositions of animal diets may partially explain the discrepancy between experiments (29).

The insignificant differences between initial weights of lambs in all treatments reflect the random distribution of animal before feeding the experimental treatments, and that any changes in the measured parameters are mainly due to the additives. One of the main objectives of the feedlot industry is to boost animal growth performance. The LAC treatment improved final body weight by 8.1%, total weight gain by 15.8% and daily gain by 15.8% compared to the control, which confirm the positive relationship observed between probiotic supplementation and animal growth performance (18, 30). As observed in the digestibility trial, LAC did not affect nutrient digestibility, which may indicate that the digestibility is not the main reason for the observed growth improvements. However, it may indicate that LAC could be recommended for growing animals while BAC, could be recommended for adult animals to improve their performance and meat quality and these will be discussed later.

The improved daily gain with probiotics supplementation to diets of ruminants are mainly due to improvement in nutrient absorption and reduction in the counts of pathogens (16). Using microbial strains improves immune system without leaving residual toxic effects (17), and this results in an improved daily gain and final body weight (18). Using additives throughout their first months of life improves gut microbiota and growth performance (11). LAC contains L. acidophilus which prevents gastrointestinal infection by pathogens or by producing antimicrobials (31, 32), and may be improved the microbial balance in the rumen of lambs compared to B. subtilis or R. albus (11), especially when be fed to animals consuming a fibrous diet (18). Increasing microbial protein synthesis with LAC additive may have increased amino acid supply at the post-ruminal level resulting in increasing daily gain (18, 33). Moreover, the results indicate that LAC increased the antagonism of pathogenic organisms through antimicrobial effects such as stimulation of host defines mechanisms and inhibition of bacterial toxins (10, 33). Additionally, probiotics increase the release of different endogenous substances, such as antibacterial substances, antioxidants, growth factors and coagulating agents, causing enhanced growth (9, 33, 34), and thereby lamb's daily gains.

The ZAD treatment decreased growth performance, which confirm the importance of selecting the suitable strain or product of probiotics. Different probiotics mean different composition, different specific activity of the probiotic strains and even different strains may have distinct effects based on enzymatic activities depending on host species (35).

Increasing growth rate without affecting feed intake in the LAC treatment was reflected as it increased feed efficiency by 11.7%, while the decreased growth rate in ZAD treatment was reflected as it decreased feed efficiency by 20.8% compared to the control. Improving feed efficiency results in improved nutrient utilization. The improved feed efficiency may be related to improved efficiency in nutrient utilization, and thereby improving N retention while reducing the excretion of essential nutrients (32). Hamdon et al. (2) observed that feeding date palm leaves treated with probiotic improved feed efficiency between 15.3 to 23.6%.

### Blood measurements

As the experiment evaluates new feed additives, blood analysis gives the opportunity to study several metabolites and other constituents that helps to detect nutritional, environmental, or physical stress. Blood components were within the ranges reported for healthy animals (21). It was expected that increasing nutrient digestibility and ruminal VFA production will increase glucose concentration, but this was not observed which may indicate that the absorption rate of glucose from treatments was not enough to be reflected in blood (36). The minor impacts of treatments on blood glucose are consistent with those observed by Abd El-Wahab et al. (16) who reported insignificantly differed blood glucose, total protein, and albumin with feeding probiotic to growing calves. However, (37) El-Mehanna et al. (37) observed an increase of 13% in blood glucose of lambs fed with probiotics. The minor effects of treatments on the concentration of blood AST and ALT, which were within the normal physiological range (38), indicate that additives did not affect liver function (39).

Additives increased the concentrations of albumin b while decreasing globulin and urea-N suggesting that they improved nutrient utilization without impairing kidney performance and protein catabolism in muscles (40). BAC and ZAD increased blood total proteins by 7.4 and 6.7%, respectively and decreased blood creatinine by 21.1 and 21.8%, respectively indicating an enhanced nutritional status of lambs and improved kidney function (38). Increased serum total protein and albumin can be related to higher nutrient supply in ewes fed with additives.

BAC, LAC and ZAD increased the concentrations of thyroid hormones (T3 by about 87.9, 73.0 and 68.8%, respectively and T4 by about 36.9, 20.9 and 32.4%, respectively). Mousa et al. (41) observed increased T3 and T4 hormones with probiotic supplementation to lambs. T3 and T4 work together to regulate energy usage and are key for controlling weight, body temperature, muscle strength, and nervous system. Thyroid hormones play important roles in the regulation of triglycerides, cholesterol metabolism, lipoprotein homeostasis, and the induction of the genes involved in glycolysis and gluconeogenesis (42). Probiotics positively affect thyroid-stimulating hormone-releasing in the hypothalamus (42).

BAC and ZAD lowered cholesterol concentration by 9.2 and 16.9%; however, El-Mehanna et al. (37) observed that probiotics supplementation to growing lambs did not affect cholesterol or insulin but increased serum glucose levels. The lowered cholesterol concentration may be due to the inhibition of cholesterol synthesis or the direct absorption of cholesterol (43). Probiotics decrease cholesterol through the deconjugation of bile acids, and the increase in the degradation of cholesterol throughout the gastrointestinal tract (44).

The increased blood hemoglobin (by 12.4, 9.4, and 10.4%, respectively), RBC (by 9.8, 8.0, and 9.8%, respectively), and MCH (by 9.4, 7.2, and 8.7%, respectively) with for BAC, LAC and ZAD treatments indicated that there was improved homeostatic impacts on the treated animals. The is scarce research on the effects of probiotics on hematological traits of animals and makes the explanation of our results challenge. These effects may be related to an increased synthesis of vitamin B12 and enhanced iron salt absorption by the small intestine, resulting in greater hematopoiesis (41). The unchanged WBC numbers with probiotic supplementation somehow indicate that animals were not under stress during the experiment (36). El-Mehanna et al. (37) observed unaffected RBC, hemoglobin levels and HCT value, while increased numbers of WBC with probiotic supplementation to growing lambs. Moreover, Sarwar et al. (45) observed increased Hb, PCV and RBC in Kajli lambs supplemented with probiotics.

### Carcass traits and meat composition

Scarce data concerning the effect of feeding *B. subtilis, L. acidophilus* or *R. albus* on carcass characteristics is available. BAC and ZAD increased fasting weight indicating anabolic effects of these additives. Such results may be due to probable increased microbial protein synthesis. Additives increased hot carcass weight, which is similar to Hamdon et al. (2) who observed improved hot carcass weight and dressing with *B. subtilis* and *Phanerochaete chrysosporium* administration to growing lambs. Increasing daily gain and body weight gain of lambs fed LAC can partially explain these results found for LAC but not in the other treatments. El-Mehanna et al. (37) observed that feeding probiotics to growing lambs did not affect carcass weight, dressing percentage and offal weights; however, increased chest depth, eye muscle area and breast weight.

The BAC treatment increased the weight of *Longissimus dorsi* muscle by 21.5%. The eye muscle area is considered as an indicator of tissue growth. Lahiri et al. (46) showed that gut microbiota impacts muscle cell metabolism across gut microbiota–skeletal muscle axis generating favorable effects in animals. Final body weight of lambs largely explains the changes in *Longissimus dorsi* characteristics (47).

BAC increased meat weight by 18.4%, while ZAD increased it by 12%. Moreover, BAC increased fat weight by 42.4%, indicating a differed ruminal fermentation and energy and fat metabolism pathways with the additives, which has been discussed earlier in this section. Increasing acetate-to-propionate ratio (48) and acetate contributes to increasing fat deposition in ruminants (49). Increased internal fat may also reflect the greater energy retention observed for those lambs that received probiotics. Crude fat is important to ensure the flavor, aroma, and juiciness of the muscle (50). Increasing the portion of fat is a result of intramuscular fat deposition, which benefits marbling, carcass quality, and isensorial properties (47).

### Nutrient digestibility and nitrogen balance

In the performance experiment, growing animals were used while adult rams were used for the digestibility experiment were and that brought differential effects that could be attributed to growth stages. Hence, data from the digestibility trial somewhat explain what was noted in the lamb trial.

The BAC treatment increased the digestibility of CP by 6.3%; however, Hamdon et al. (2) observed lowered CP digestibility with feeding lambs fed on a diet containing date palm leaves supplemented with microbial feed additives. They explained that results due to the presence of a substantial amount of CP related to the fiber fraction, which lowered disposal of these proteins for the animal (51). Probiotic type and grinding degree of date palm leaves may have been different between the one used in their experiment contrasted to the one applied in the present study, and this may partially explain the difference between their experiment and ours.

It was expected that the presence of high concentrations of carotenoids, isoflavones, lignans, flavonoids, tannins, and sterols in ate palm leaves can negatively affect nutrient digestibility (52); however, these effects were not observed as the values of nutrient digestibility were within the ranges reported for ruminants. Such results indicate the ability of the evaluated additives to cover the negative effects of bioactive components on nutrient digestion. The BAC and ZAD improved digestibility of DM (by 7.8 and 5.5%, respectively), OM (by 8.2 and 6.9%, respectively) and NDF (by 14.1 and 10.7%, respectively), while BAC, LAC and ZAD increased digestibility of NSC by 9.2, 9.1 and 8.5%, respectively. Many modes of action were proposed to explain these results (11, 12). In the present experiment, the improved CP and fiber digestibility indicate that feeding microbial feed additives may loosen the association among the fiber bundles and between protein and fiber fractions in date palm leaves (2). Although activity of ruminal cellulolytic microbial populations was not measured in the present experiment, microbial supplementation could produce a tonic level of lactate, which will then boost a basal abundance of lactate utilizing bacteria in the rumen, thus stabilization of pH as observed in the metabolism trial in the present experiment (8, 32). The improved ruminal environment may increase the fiber degrading microbial communities in the rumen resulting in improved nutrient digestion and synthesis of microbial proteins (15). Furthermore, improved digestibility may enhance the interaction of microbes in the additives with the ruminal microbial flora (53, 54) and increase enzyme activity in the gastrointestinal tract resulting in improved nutrient digestibility (55, 56). Another reason for the improved nutrient digestibility is the probable beneficial inhibitory effects of probiotics on the gastrointestinal infection by pathogens (32). Additionally, experiments (57) showed that probiotic supplementation improves ruminal cellulolytic microbial populations and buffers rumen pH, leading to enhanced nutrient digestion (33) and synthesis of microbial proteins (15).

Additives decreased fecal N by 11.7, 9.2, and 8.6% and increased N balance by 9.3, 7.7, and 8.8% for BAC, LAC and ZAD, respectively indicating improved N utilization by animals. Sallam et al. (18) observed improved N balance by 22% when animals were fed a diet supplemented with probiotics. Moreover, Kawauchi et al. (58) observed that feeding cows on a diet supplemented with B. subtilis did not affect the amounts of N loss in the feces or urine or retained N. Increased N retention in body tissues may explain the increased daily gain in animals fed additives. Lack of differences in ruminal ammonia-N, blood urea-N concentrations and N excreted revealed an improved utilization of nutrients (59). These effects are desirable from an environmental point of view as N is rapidly mineralized from NH3/NH4+ to nitrous oxide, which has a huge global warming potential of about 265–298 compared with 21 for methane.

### Ruminal fermentation

Rumen pH (60) and ammonia-N (61) were greater than rates required for fiber digestion and microbial protein synthesis. Probiotics did not impact rumen pH, and this is important to avoid changes of rumen microbial population from fibrolytic to amylolytic microbes (62). Probiotics work effectively to balance ruminal pH (11, 12). In the present experiment, the values of ruminal pH indicate that lambs kept their rumen near neutrality (6.84–7.04), which may be related to feeding lambs on a fibrous diet based on date palm leaves. Abd El-Wahab et al. (16) observed no effects on ruminal pH of growing calves fed probiotics.

Probiotics did not impact ammonia N, indicating minimal effects on dietary protein breakdown and digestion, and the uptake of ammonia by bacteria (58). However, BAC increased CP digestibility indicating increased uptake of ammonia by bacteria and increased microbial protein in the rumen. Such assumption may be partially explaining the increased meat proportion in the carcass of lambs fed BAC. The rumen microflora starts to convert a part of ammonia-N into microbial proteins, which represent an essential source of N for the growing animal, and another part is recycled as urea (63). Similarly, Kawauchi et al. (58) observed minor effects on ruminal ammonia-N concentration when feeding B. subtilis to cows.

The BAC and ZAD increased ruminal VFA concentration by 33 and 9.9%, respectively, which may be related to an increased digestibility of OM, NSC and NDF. Increasing microbial population can result in increased VFA with positive impacts on energy supply to the animal (32). Increasing the production of VFA ensures enough energy formation and stimulates the growth in the surface, length, and width of rumen papillae (64). Increased total VFA concentration likely resulted from the increased fermentable carbohydrate availability, ruminal microbial activity, and fermentation rate.

## Conclusions

In arid environments, date palm leaves can be utilized as forage source for lambs. However, improving its utilization with probiotic additives is recommended. The responses to different products of probiotics containing different types and proportions of microbes differ. Probiotics positively affected blood chemistry and improved N balance and carcass characteristics. Bacillofort increased ruminal volatile fatty acids production and improve meat quality in lambs as increasing the weight of *Longissimus dorsi* muscle, meat, and fat. However, Lacotpro could be used to improve growth performance and feed efficiency.

## Data availability statement

The raw data supporting the conclusions of this article will be made available by the authors, without undue reservation.

## Ethics statement

The protocol of the experiment was reviewed and approved by the Institutional Animal Care and Use Committee of the Faculty of Agriculture, New Valley University, New Valley, Egypt.

## Author contributions

Conceptualization: HH, AYK, GA, TS, MF, and AEK. Methodology, investigation, and resources: HH, AYK, GA, TS, and MF. Validation and data curation: HH and AEK. Project administration: HH and AYK. Formal analysis: HH, TS, and AEK. Visualization: HH, TS, and AYK. Supervision: HH, AYK, GA, and MF. Writing—original draft preparation: AEK. Writing—review and editing: EV-B-P and AEK. All authors have read and agreed to the published version of the manuscript.

## Conflict of interest

The authors declare that the research was conducted in the absence of any commercial or financial relationships that could be construed as a potential conflict of interest.

## Publisher's note

All claims expressed in this article are solely those of the authors and do not necessarily represent those of their affiliated organizations, or those of the publisher, the editors and the reviewers. Any product that may be evaluated in this article, or claim that may be made by its manufacturer, is not guaranteed or endorsed by the publisher.
